# Dynamic Assembly and Multifunctional Roles of Plant Endophytic Microbiomes

**DOI:** 10.3390/microorganisms14092118

**Published:** 2026-09-21

**Authors:** Jie Song, Wenhui Liu, Yan Sun, Jinfeng Shi, Meiqi Liu, Yuting Dai, Yuhu Zuo, Qin Yao

**Affiliations:** 1 College of Agriculture, Heilongjiang Bayi Agricultural University, Daqing 163319, China; songjiend@163.com (J.S.); 19586144095@163.com (W.L.); 18245735278@163.com (J.S.); 18763950677@163.com (M.L.); 19586163925@163.com (Y.D.); zuoyhu@163.com (Y.Z.); 2 Heilongjiang Provincial Key Laboratory Crop Pest Interaction Biology and Ecological Control, Daqing 163319, China; 3 National Coarse Cereals Engineering Research Center, Daqing 163319, China; 4 School of Resources and Environment, Northeast Agricultural University, Harbin 150030, China; sunyan0625@neau.edu.cn; 5 Engineering Research Center of Crop Straw Utilization, Heilongjiang Province, Daqing 163319, China; 6 Key Laboratory of Low-Carbon Green Agriculture in Northeastern China, Ministry of Agriculture and Rural Affairs P.R., Daqing 163319, China

**Keywords:** endophytic microbiome, community assembly, plant–microbe interactions, plant growth promotion, stress tolerance

## Abstract

Healthy host plants harbor taxonomically structured and diverse endophytic microbial communities that establish sophisticated symbiotic crosstalk with their hosts. These endophytic microbiomes confer multiple beneficial traits, including growth promotion, nutrient acquisition, and enhanced resistance to biotic and abiotic stresses, and are increasingly recognized as key modulators of plant fitness. The assembly of endophytic communities is not random but shaped by combined effects of environmental cues, host filtering, and microbial–microbial interactions, among which plant immunity constitutes an important host-selection dimension. Beneficial endophytes deploy diverse molecular tactics, such as masking microbe-associated molecular patterns (MAMPs) and secreting immune-suppressive compounds, to evade host PAMP-triggered immunity and effector-triggered immunity (PTI-ETI) surveillance for persistent internal colonization. In this review, based on the literature retrieved from Web of Science Core Collection and Scopus (2010–2026), we systematically summarize the colonization process, dynamic assembly rules, and driving factors of plant endophytic microbiomes. We further elaborate their multifaceted physiological functions in regulating plant growth, nutrient utilization, and stress adaptation. Deciphering such multilayered plant–endophyte interactions provides important insights for harnessing beneficial endophytes to advance sustainable agricultural development.

## 1. Introduction

In natural environments, plants provide diverse ecological niches for a large variety of microorganisms [1]. Endophytic microbes establish intricate symbiotic relationships with host plants. The co-evolution of plants and microorganisms plays a critical role in maintaining ecosystem stability. Endophytic microbes establish intricate symbiotic relationships within plant tissues and substantially benefit host performance, including growth promotion, nutrient uptake, stress tolerance and pathogen resistance [2,3]. The assembly of plant-associated endophytic microbial communities is not a random process, but follows the general ecological rules [4,5,6]. This assembly is mainly shaped by complex interactions among microbial communities, host-derived selective pressures, and external environmental factors. However, the ecological and evolutionary mechanisms governing host-mediated assembly of plant endophytic microbiomes remain incompletely understood. Therefore, exploring the assembly rules and functional outputs of plant-endophyte communities is of great significance for improving plant adaptability and facilitating microbial application in agricultural ecosystems.

Healthy plants possess highly diverse yet taxonomically structured endophytic microbial communities. These microorganisms can establish stable and consistent associations with specific hosts and play a vital role in maintaining plant physiological function and health [7,8,9,10]. The core microbial communities provide direct beneficial effects for host plants [11,12,13]. They assist hosts in coping with adverse environments by transforming and translocating essential soil nutrients, and defend against phytopathogens through competition, antibiosis, and secretion of hydrolytic enzymes [14]. In addition, under distinct environmental conditions, endophytic community assembly indirectly modulates plant resistance responses [15,16]. Therefore, deciphering evolutionary and ecological mechanisms driving endophytic community assembly is critical for improving plant adaptability and productivity. Such knowledge also facilitates the practical applications of beneficial microorganisms in agricultural ecosystems.

In this review, we systematically synthesize current knowledge on the complex interplay between host plants and their endophytic microorganisms. We further elaborate on the dynamic assembly of endophytic communities, which is driven by multilayered host-filtering mechanisms and fluctuating environmental conditions. We also characterize the diverse functions of these assembled microbial communities. Deciphering such plant–microbe interactions provides new insights into endophyte research, and offers valuable guidance for sustaining plant health, enhancing host stress adaptability, and boosting crop productivity within sustainable agriculture. Despite the rapidly accumulating sequencing-based descriptive data on plant endophytic communities, substantial mechanistic gaps persist between observational community profiles and practical agricultural translation. Many previous studies largely relied on datasets to characterize endophytic microbiome patterns, while direct experimental validation of causal links between community assembly, microbial metabolic potentials and host physiological performance remains limited. Furthermore, most existing reviews tend to separately elaborate on either community assembly rules or microbe-mediated beneficial traits. Integrative overviews that systematically connect dynamic assembly processes with the diverse functional outputs of endophytic microbiomes are relatively scarce. Therefore, this review synthesizes current progress covering both assembly mechanisms and multifunctional roles of endophytic microbiomes. We also highlight unresolved bottlenecks restricting the application of beneficial endophytes to provide theoretical references for follow-up mechanistic studies and the development of microbe-assisted agricultural strategies.

To collect and summarize published evidence for this review, we performed a targeted literature search. Relevant publications were retrieved from Web of Science Core Collection and Scopus, with Google Scholar used as a supplementary source. The literature search covered publications from 2010 to 2026, and the final retrieval was completed in June 2026. Core search keywords included endophytic bacteria, endophyte, plant–microbe interaction, root microbiome, colonization, plant immunity, and nutrient mobilization.

## 2. Symbiotic Relationship of Plants and Endophytic Microorganisms

**Figure 1:**
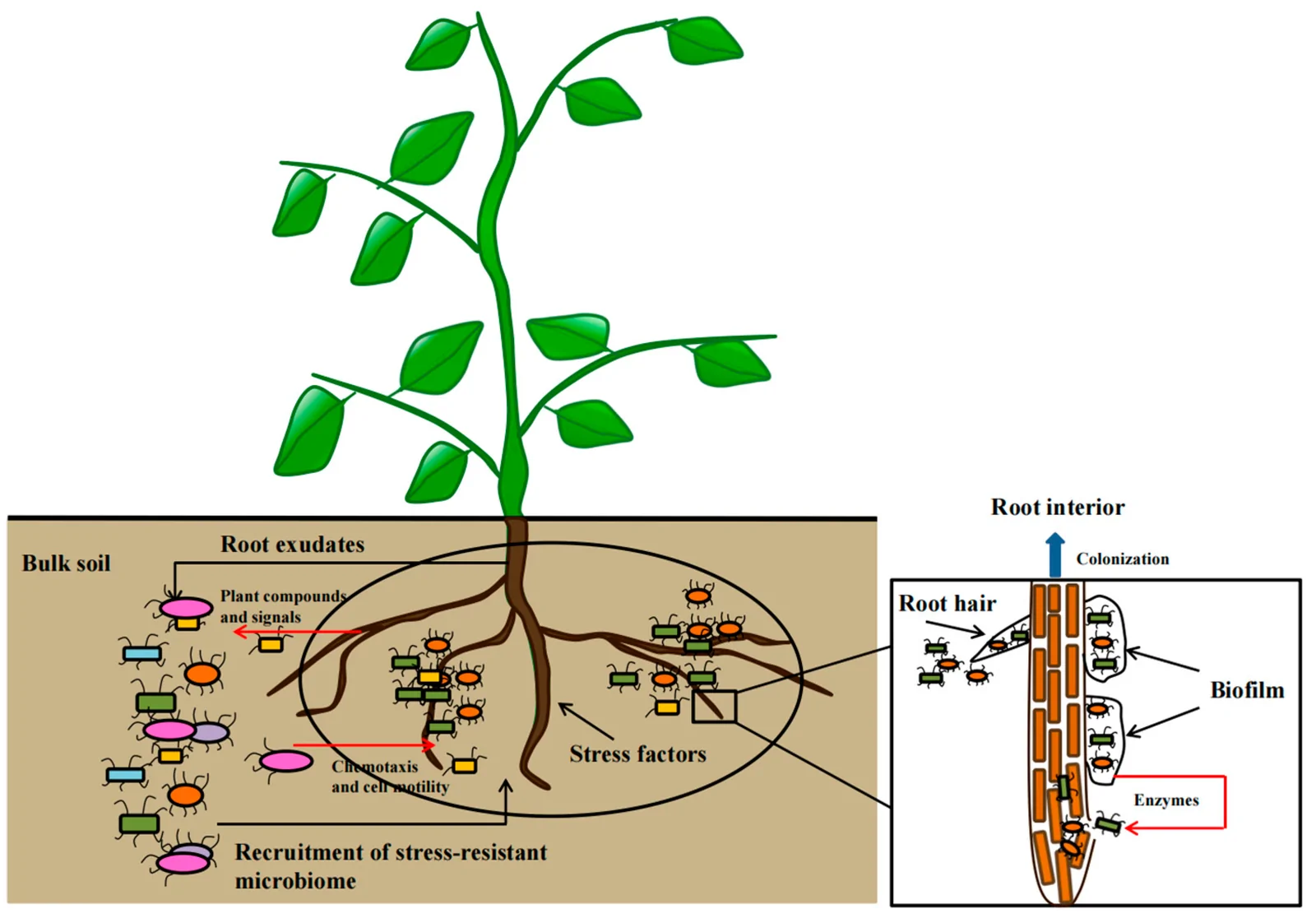
Schematic representation of diverse and complex interactions between plants and plant-associated microbiomes. Soil microbiota is represented by ovals and rectangles. Specifically, yellow, green and orange denote beneficial microbiota, while pink, blue and light purple represent other non-beneficial soil microorganisms. The panel inset shows a magnified view of the boxed region in the main figure.

Plants establish intimate symbiotic associations with diverse endophytic microorganisms under natural conditions [17]. These microorganisms participate in plant physiological processes and diverse metabolic activities, and the plant-associated microbiome co-regulates multiple host-plant traits [18,19,20]. They play vital roles in regulating plant growth and functional performance (Figure 1). Therefore, plants and their associated microbiota constitute a ‘holobiont’, where interactions occur between plants and microorganisms [21,22,23,24,25]. Endophytic bacteria, as a crucial component of plant-associated microbiomes, reside inside healthy plant tissues without causing external infection symptoms [26]. Plants and endophytic bacteria establish a mutualistic symbiotic relationship during long-term co-evolution [27,28,29,30]. Host plants supply abundant essential nutrients for the growth of endophytic bacteria; in return, endophytic bacteria exert multiple biological functions, including promoting plant growth and reproduction, enhancing host disease resistance, and accelerating wound repair. These endophyte-mediated effects ultimately improve the adaptive fitness of host plants [31,32,33,34,35,36].

Colonization of endophytic bacteria in plants is an extremely complex process [37]. Endophytic bacteria sense and respond to phytogenic signals, such as organic acids or sugars, in plant secretions via chemotaxis, and migrate toward plant tissues using flagella (Figure 1). Perception of these molecules triggers lifestyle-related changes in endophytic bacteria, which further facilitate the attachment of these colonizers to root surfaces and subsequent biofilm formation [34,38,39]. This is the initial stage of endophytic colonization, during which bacteria search for entry sites on the host plant [40]. Endophytic bacteria secrete cellulase to degrade plant cell walls, which in most cases primarily enables microbial access to apoplastic intercellular spaces rather than full penetration into host cells [41]. The extent of cell-wall degradation determines whether microbes reside within cell-wall matrices, occupy apoplastic compartments, enter intracellular spaces, or spread systemically through vascular tissues. Microbes may also colonize the root cortex, vascular tissues, and intercellular spaces via roots and root hairs. Successful establishment of colonization allows these endophytic colonizers to proliferate within the nutrient-rich microenvironment provided by host plants [42,43]. To maintain persistent colonization inside plant tissues, endophytes must evade or temper host immune surveillance. Beneficial endophytes have evolved multiple strategies to avoid excessive host immune activation. They mask microbe-associated molecular patterns (MAMPs) and secrete multiple bioactive molecules, including immune-suppressive effectors, phytohormone mimics, and volatile compounds. These compounds dampen both PAMP-triggered immunity (PTI) and effector-triggered immunity (ETI) responses [16,21]. Without such modulatory tactics, endophytes would be recognized and eliminated by plant-defence systems.

Within this symbiosis, endophytic bacteria interact with their host plants and, in turn, modulate host growth and development by producing diverse bioactive metabolites and mediating nutrient transformation [44]. Such regulatory outputs are mediated by diverse inter-kingdom communication molecules rather than simple nutrient exchange. Endophytic bacteria secrete bioactive compounds, including phytohormones, volatile organic compounds and the functional enzyme 1-aminocyclopropane-1-carboxylate (ACC) deaminase. Bacterial small-RNA effectors can be translocated into host cells to alter plant gene expression. In turn, plants secrete root exudates, phytohormones and small RNAs to remodel endophytic community assembly [45,46,47]. These chemical and molecular dialogues constitute the core molecular basis for the reciprocal plant–endophyte crosstalk beyond simple nutrient supply. Notably, successful colonization of host tissues by endophytic microorganisms can reshape the assembly dynamics of plant-associated microbial communities [30]. For example, endophytic colonization can trigger activation of plant hormone- and nutrient-sensing signaling pathways, or cause root-growth modification [48]. These physiological changes subsequently alter host root architecture and shift the niche-colonization patterns of diverse endophytic microbial communities. Collectively, these multilayered processes drive the establishment of endophytic communities.

Such reciprocal chemical communication also drives complex growth–defence trade-offs during plant–endophyte symbiosis [46]. To restrict over-proliferation of endophytic microbes inside plant tissues, host plants often maintain a basal level of immune activity even when colonized by beneficial symbionts. Complete suppression of plant immunity would allow unrestricted microbial multiplication, which may turn formerly beneficial endophytes into opportunistic pathogens under certain conditions. Therefore, a balanced immune output is critical to sustain mutualistic symbiosis. On the microbial side, beneficial endophytes must fine-tune their metabolic activity and proliferation rate inside host tissues to avoid triggering excessive host immune responses [21]. Uncontrolled bacterial growth within apoplastic spaces can trigger strong defence reactions and lead to clearance of these symbionts [41]. This delicate balance implies that mutualism is not a fixed trait but a context-dependent state, which can shift along environmental gradients, host developmental stages, and microbial population density. Understanding how plants and endophytes dynamically tune this growth–defence equilibrium helps explain why the same microbial strain can exert divergent effects on host performance under different conditions. It also provides further mechanistic insight into the variable endophyte–host interaction outcomes observed in greenhouse and field settings.

## 3. Dynamic Construction of Plant Endophytic Microbial Communities

**Figure 2:**
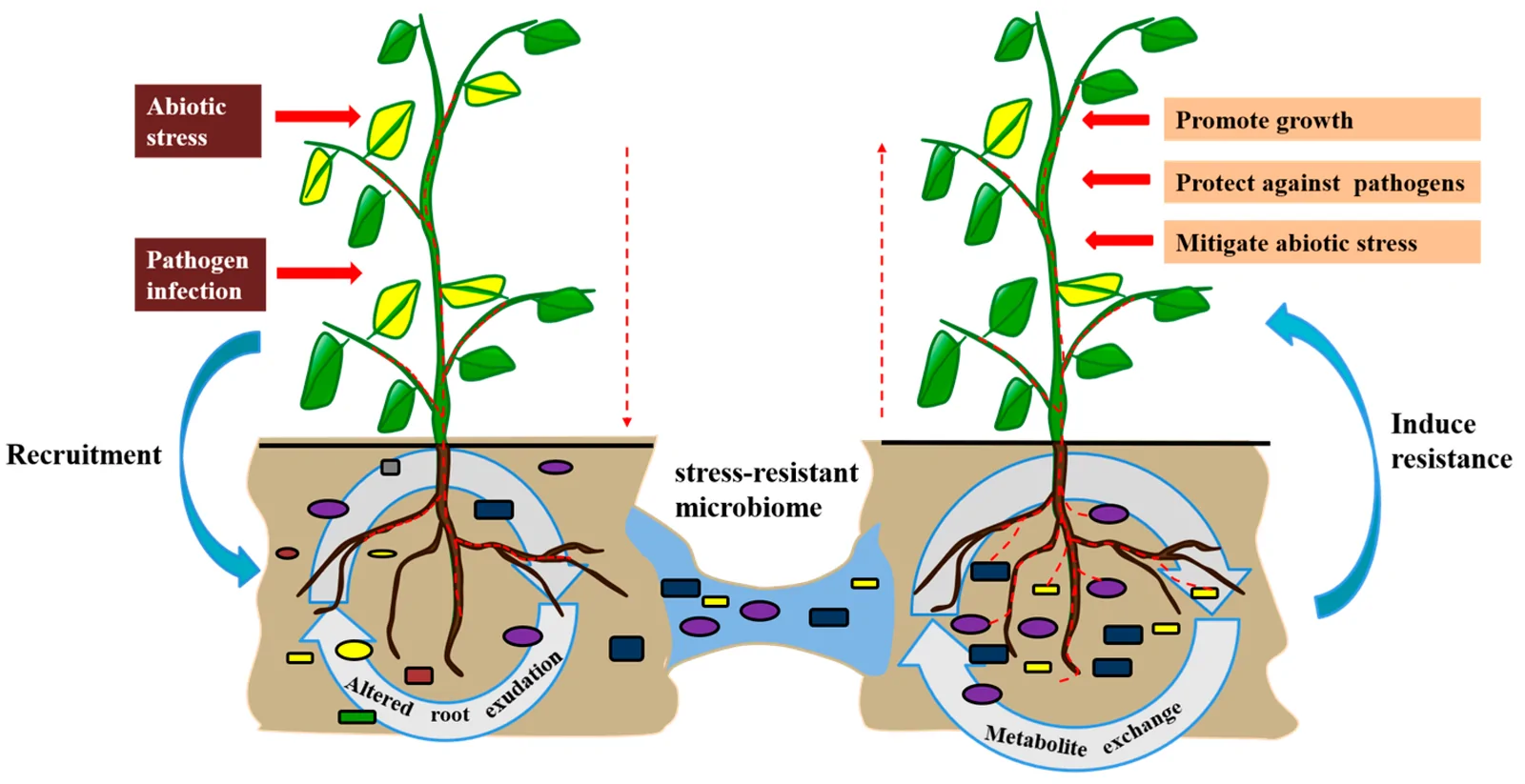
Schematic illustration of dynamic assembly of plant endophytic microbial communities. The left panel illustrates plant–microbiome interactions under different stress conditions. The right panel illustrates the functions of dynamically recruited microbial communities.

Plant-associated microbial communities, particularly endophytic consortia, exhibit distinct phylogenetic structures [49]. Endophytic community assembly is jointly shaped by complex interactions among microorganisms, plant hosts, and environmental variables. Soil and air supply diverse habitats for plant- associated microbiome, supplying potential colonizers for endophytic niches [50,51]. The microbiome of above-ground plant tissues is susceptible to material transport processes, whereas below-ground microbial communities are shaped by multiple factors including soil type, nutrient availability, and water conditions [50,51,52,53]. Microbes experience strong regional filtering and different plants offer unique ecological niches for their endophytes, which further modulates the assembly of endophytic microbial communities [54]. Therefore, endophytic microbial communities are not static, and their diversity exhibits strong dynamics throughout the lifecycle of the plant host (Figure 2). The assembly of plant-associated microbiomes shifts gradually from bulk soil to the rhizosphere and then to endophytic compartments, with microbial abundance declining along this gradient while microbial diversity increases [55]. This spatial gradient highlights the vital role of host-filtering effects in shaping endophytic community composition [56,57]. Microbial communities undergo habitat filtering over the temporal scale of plant development, and the host-specific selection filtering imposed by plants gradually intensifies along the gradient from bulk soil and rhizosphere to the root–shoot interface [58,59,60,61]. Plants provide a suitable growth environment for recruited microbes, thereby enriching key microbial populations.

The dynamic assembly of endophytic communities further delivers multiple fitness benefits for host plants under biotic and abiotic stress conditions. Dynamic assembly of plant microbiomes benefits plant health and defends hosts against exogenous damage under biotic and abiotic stress conditions [21]. It has been reported that arbuscular mycorrhizal fungi and nitrogen-fixing rhizobia, which can occupy internal plant tissues as endophytes, absorb and transport nutrients for hosts under low-phosphorus or low-nitrogen conditions [62]. Under pathogen infection, plants can reshape their root microbiota and specifically recruit a suite of disease-suppressive and growth-promoting beneficial microbes, some of which can further establish endophytic residence, to improve the survival rate of their offspring in the same soil [63]. When *Arabidopsis thaliana* was infected by downy mildew pathogens, three microbial strains were recruited to form a synergistic consortium that jointly induced plant systemic resistance against downy mildew and promoted plant growth [2]. It indicates that microorganisms colonizing identical host ecological niches not only compete for limited resources, but also participate in microbial assembly. Under given environmental conditions, plants mediate the recruitment of specific microbes to assemble core endophytic microbiota. These core members maintain high relative abundances, thereby alleviating adverse environmental stress. An emerging view holds that interactions between plants and their endophytic microbiomes can shape novel plant phenotypes with enhanced fitness under distinct environmental conditions [64]. These endophytic microorganisms possess multiple functional characteristics, including improving host resistance to pathogens, modulating plant immunity, establishing defense systems, and co-regulating host–microbiome interactions.

Despite accumulating correlative evidence for stress-triggered microbial recruitment, several critical limitations remain to be addressed. Specifically, most relevant observations are solely derived from community-sequencing datasets [2,63,65]. Shifts in microbial relative abundance alone cannot confirm that recruited taxa, including potential endophytic members, deliver genuine beneficial functions within plant tissues. Even when functional capacities of these microbes are validated under controlled experimental settings, their beneficial effects are not always reproducible under complex field conditions. Diverse native soil microbial backgrounds, fluctuating environmental factors, and multiple co-occurring stresses can interfere with the activity of recruited microbes. In addition, potential growth–defense trade-offs merit consideration: defense activation triggered by recruited endophytic taxa may impose fitness costs on host-plant growth. Therefore, more evidence is required to verify whether stress-enriched endophytic taxa can stably colonize and consistently deliver beneficial functions under real-world agricultural scenarios.

**Figure 3:**
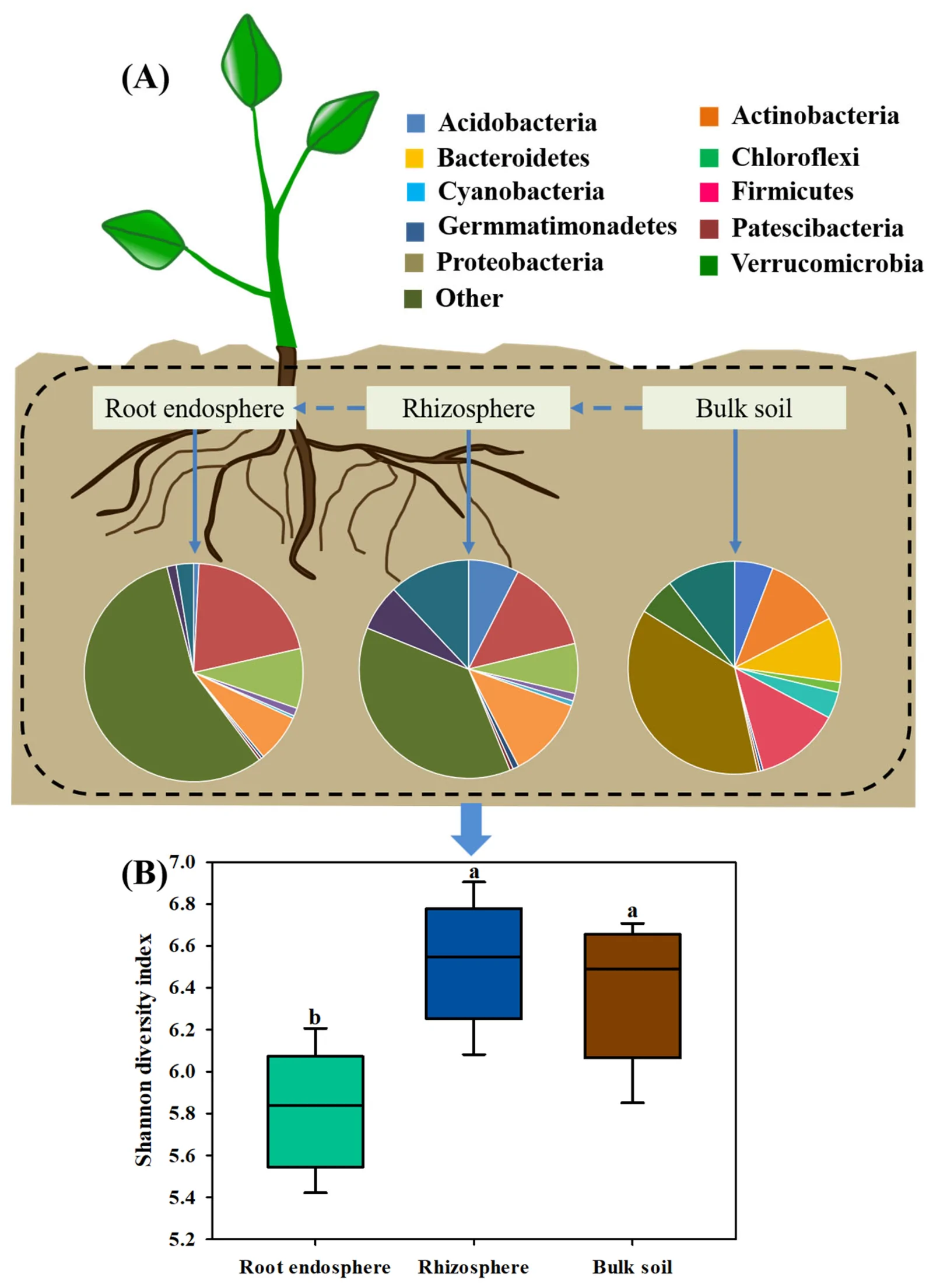
Bacterial community structure in maize root endosphere, rhizosphere and bulk soil. Data derived from Ref. [76]. Panel (**A**): Relative abundances of the dominant bacterial phyla across bulk soil, rhizosphere and maize root endosphere. Panel (**B**): Bacterial α-diversity calculated as the Shannon index for bulk soil, rhizosphere and root endosphere. Different lowercase letters denote significant differences among compartments (*p* < 0.05).

Host-imposed selective filtering represents the core mechanism shaping endophytic assembly along the soil–plant continuum. The co-evolution of plants and their associated microbiomes is modulated by environmental conditions and host selection (Figure 3). Plants provide a variety of microhabitats for microorganisms, and successful colonization of endophytic niches requires microbes to possess the capacity to tolerate stresses derived from plant immune responses and microhabitat characteristics [66,67,68]. Tolerance or evasion of host-immune-derived selective pressure is a prerequisite not only for pathogenic infection but also for successful colonization of plant internal niches by endophytic microorganisms [69,70]. Under different environmental conditions, plants secrete a suite of signaling-related metabolites, such as organic acids, phenolics and soluble sugars, to attract target microorganisms. Plants further amplify this selective process either by tuning host immune output or by supplying specialized nutritional microhabitats, thereby recruiting beneficial endophytic taxa [69,71,72]. Plants synthesize diverse metabolites, and multiple core-function microorganisms can enhance the biosynthesis of these compounds in host plants [73,74,75]. Key metabolites, including organic acids, sugars, phenolics, and peroxidases, are secreted into the plant apoplast and intercellular spaces, supplying carbon-rich substrates for endophytic populations to sustain their *in planta* growth and survival. Selected microorganisms colonize and accumulate inside plant tissues as endophytes after sensing host-derived signal molecules, while other microbial taxa are filtered out (Figure 3). Notably, such host-driven microbial recruitment is not universal. It is strongly shaped by soil microbial reservoirs, plant genotype, developmental stage, as well as abiotic environmental conditions. For example, multiple Andean maize genotypes assembled consistent core bacterial ASVs under greenhouse conditions with local agricultural soil, yet these patterns can differ in the field or under distinct soil backgrounds [76]. Studies have demonstrated that along the soil–plant continuum, endophytic community composition is primarily shaped by the host-derived niches and crop species, whereas geographic location and fertilization regimes exert weaker effects [77,78,79,80]. From the soil to the plant surface and subsequently to the plant interior, the host selection effect gradually increases, while the diversity and network complexity of the bacterial community decline [53,81]. Crop microbiomes are primarily derived from the soil environment and gradually accumulate while being filtered across distinct plant spatial niches. Notably, community composition and functional potential differ substantially across plant compartments, as roots, stems, leaves and seed tissues harbor distinct endophytic microbiota. Within seeds, endophytes can occupy discrete niches such as the seed coat, endosperm and embryo. These compartments largely determine the routes for vertical microbial transmission. Endophytic assemblages are shaped by compartment-specific selective pressures, yet a small set of core taxa can still be detected consistently across these niches. Beyond tissue-driven variation, host species also impose strong selection for distinct endophytic taxa. For example, *Bacilli* and *Methylobacteriaceae* are identified as key biomarker taxa of barley, wheat and maize, respectively [24].

However, how host and environmental factors drive this selective filtering and mediate endophytic microbial community assembly remains incompletely resolved. Metagenomic approaches help uncover key molecular determinants driving community assembly. For instance, genomic analysis of plant-associated microbes can identify genes for carbohydrate metabolism, transport systems and corresponding transcriptional regulators. These gene products support microbial carbon acquisition and compartment-specific colonization within plant tissues. Beyond enabling microbial colonization, such microbial metabolic activities generate bioactive metabolites and modify *in planta* nutrient status. These changes, in turn, reshape host-plant fitness and physiological performance. Such multi-omics findings provide valuable insights into how microbes within endophytic communities modulate host physiological functions. Importantly, host-genotype-driven filtering of endophytic microbiomes is frequently modulated by genotype-by-environment interactions. Strong host-genotype-specific microbiome patterns observed under controlled greenhouse conditions are often attenuated under complex field environments, where fluctuating temperature, water availability, and native soil microbial pools collectively reshape microbial recruitment. Even different cultivars within the same crop species can assemble distinct endophytic consortia, which partially explains why beneficial microbial inoculants frequently exhibit inconsistent performance across field sites [49]. Such context-dependent host-filtering effects constitute a major bottleneck when translating laboratory-derived knowledge into practical agricultural applications. Therefore, evaluating host–microbe interactions across multiple field conditions is essential prior to deploying endophyte-based biostimulants.

## 4. Functions of Plant Endophytic Microorganisms

**Figure 4:**
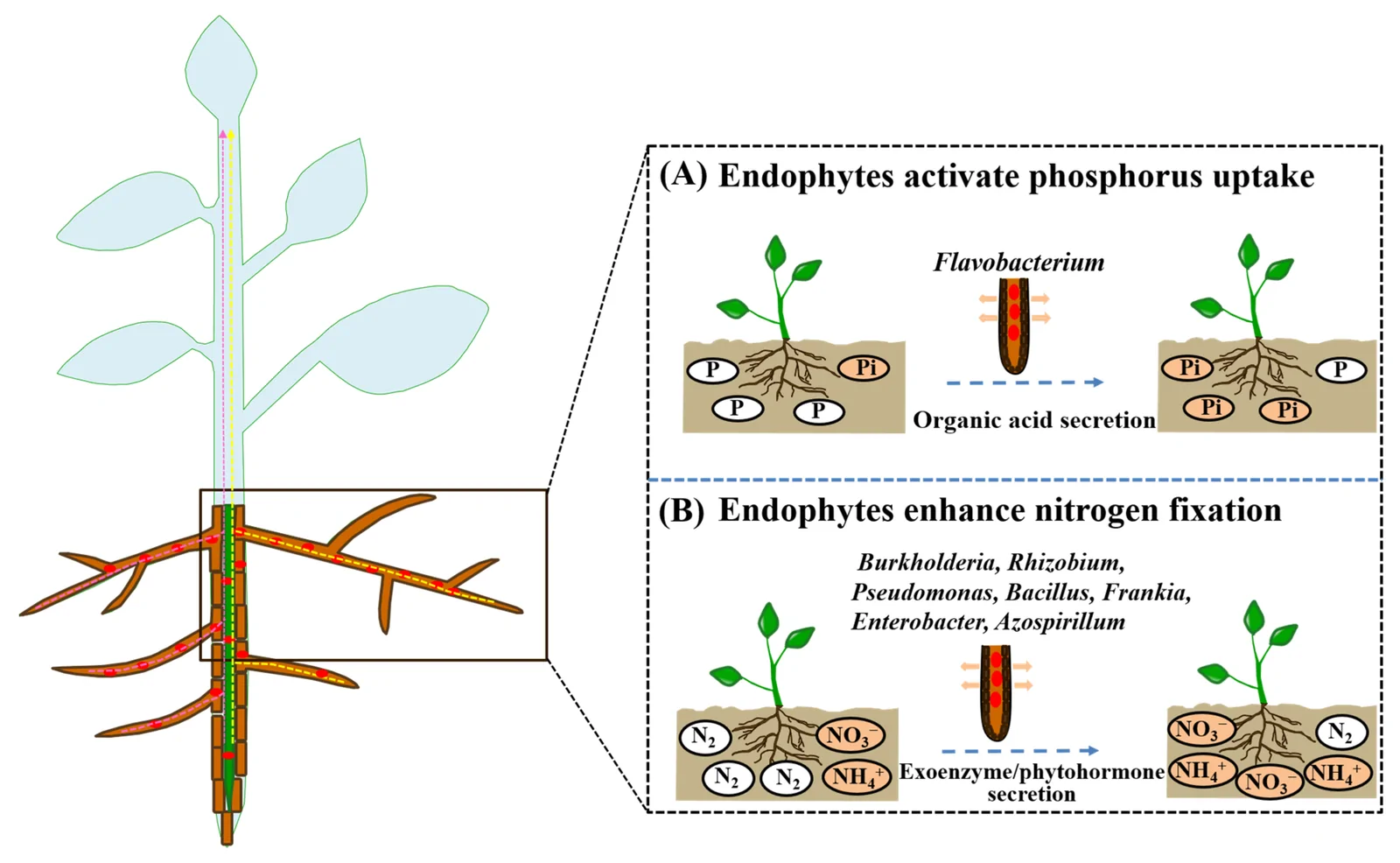
Functional community structure of plant endophytic microbiota. Panels (**A**,**B**) are magnified views of the boxed regions in the main panel. (**A**), Endophytes activate phosphorus uptake; (**B**), Endophytes enhance nitrogen fixation. Note: This diagram mainly illustrates bacterial-mediated nutrient processes; fungal endophytes also participate in phosphorus uptake, as described in the main text.

Plant endophytic microbial communities play a critical role in plant growth via multiple mechanisms, including increasing nutrient access, stimulating phytohormone synthesis, and defending hosts against pathogens [49,81,82,83]. Nutrient acquisition and stress-immune adaptation represent two cores, tightly coupled functions of endophytic microbiomes. Together, they regulate plant fitness and modulate endophytic community assembly. For stress-immune adaptation, endophytes can trigger plant-defence signaling cascades, synthesize protective compatible solutes and detoxify harmful reactive oxygen species to mitigate stress-related damage. Nutrient availability acts as a fundamental environmental filter driving the recruitment and colonization of core endophytes, while immune responses further refine endophytic community structure under biotic stress. Some plant growth-promoting bacteria enhance root water and mineral uptake via multiple mechanisms. They secrete organic acids and phosphatases to solubilize sparingly soluble phosphorus, and produce siderophores that chelate iron, mobilize phosphate from iron-phosphate complexes, and solubilize copper (Cu) and zinc (Zn) [45,49]. They also release auxins to stimulate lateral root and root hair development, thereby remodeling host-plant root architecture. Microbial communities assembled in plants can mobilize nutrients that are not readily available to plants, to meet plant demand for essential elements [84,85,86,87]. Some studies have indicated that differences in nitrogen-use efficiency across rice varieties are mainly driven by microorganisms involved in the nitrogen cycle [88,89,90]. These microorganisms mineralize organic nitrogen into ammonium nitrogen, which is subsequently oxidized to nitrate via nitrification. Such sequential nitrogen transformations further enhance the nitrogen-use efficiency of rice plants [90,91]. Under low-inorganic-phosphate conditions, synthetic plant–microbe consortia upregulate plant phosphate-starvation responsive genes. These artificial microbial assemblies enhance plant phosphate assimilation and phosphorus uptake, thereby promoting plant growth in phosphate-deficient soils [6,91,92,93,94,95]. Besides bacterial endophytes, fungal endophytes (e.g., arbuscular mycorrhizal fungi) also substantially contribute to plant phosphorus acquisition. These symbiotic fungi extend their extraradical hyphae into soil compartments, expand the soil-exploring area of host roots, and mobilize sparingly soluble phosphorus resources for plant uptake [73]. This interplay between plant nutritional status and its associated microbiomes shapes the assembly of beneficial functional microbial communities, highlighting the critical role of endophytic microbiomes in regulating diverse plant physiological functions (Figure 4).

Despite promising plant-beneficial effects observed under controlled laboratory or greenhouse conditions, the practical performance of single-strain inoculants and synthetic microbial consortia is often variable under field conditions. Native soil microbial communities exert strong competitive and antagonistic pressure on introduced microbes, which frequently fail to achieve stable endophytic colonization inside plant tissues [30]. Soil physicochemical properties, background microbiome composition, crop cultivars, and fluctuating abiotic conditions collectively determine whether inoculated strains can persist and exert expected nutritional functions. Even well-characterized beneficial endophytes may lose their plant-growth-promoting capacity in complex field soils. This phenomenon underscores the gap between mechanistic laboratory studies and real-world agricultural implementation. Improving the ecological fitness of inoculated microbes, optimizing application strategies, and matching microbial candidates to local soil-crop systems are therefore essential steps to realize the agricultural potential of beneficial endophytes. Beyond nutrient-related physiological adjustments, host plants employ immune signaling as another critical regulatory layer to govern endophytic community assembly. At the molecular level, plant innate immunity consists of two layered defence tiers: PAMP-triggered immunity (PTI) and effector-triggered immunity (ETI). Endophytic microorganisms can modulate both PTI and ETI during host colonization [96,97]. Certain endophytes trigger mild PTI signaling to prime host defence without triggering severe growth–defence trade-offs, whereas compatible endophytic symbionts may fine-tune ETI outputs to sustain stable internal colonization. This bidirectional immune crosstalk facilitates endophytic persistence and serves as a key host-selection filter shaping resident endophytic community assembly. Such immune crosstalk also improves host stress-immune performance. At the community level, these immune-driven recruitment processes further shape plant–microbe interactions under stress conditions.

Building on the above mechanistic descriptions, host root immune responses reshape root exudation profiles to recruit beneficial rhizosphere microorganisms and activate systemic resistance [98,99,100,101]. This constitutes a well-documented plant defensive strategy. When pathogens breach the rhizosphere defense barriers, the endophytic microbiome can selectively enrich specific microbial members to produce enzymes and secondary metabolites that inhibit pathogens and induce plant systemic resistance [75,102,103,104]. Some studies have shown that tomato can selectively recruit endophytic disease-resistant microbial communities, which enhances host disease resistance and alleviates damage caused by fusarium wilt [14]. Consistently, plants can recruit and enrich stress-resistant endophytic microbial communities under biotic or abiotic stress and relieve stress by modulating plant hormone production and host biochemical activity [105,106,107]. Some endophytic communities can also modulate host phenylpropanoid pathways and salicylic acid biosynthesis. These microbes reinforce the plant cell wall and boost host resistance against pathogen infection and abiotic stress [7,31,73,76]. Stress tolerance and immunity exhibit such direct interplay. Combined with selective recruitment and enrichment of beneficial endophytic communities, this process substantially improves plant adaptability under stressful environments [108,109,110,111]. However, further studies are still required to elucidate plant–microbe interactions and their responses to environmental conditions across different crop species.

## 5. Conclusions and Future Perspectives

Extensive surveys of key crops and plant species have generated preliminary insights into their dominant bacterial and fungal endophytes. Nevertheless, substantial plant-associated microbial diversity, core microbiome assemblies, and the host physiological functions mediated by these microbiomes remain to be uncovered in most plant species. Systematic frameworks for profiling microbiomes of ecologically and economically valuable plants are urgently required to characterize core endophytic microbiomes and the beneficial host functions they confer. Such standardized strategies enable comprehensive assessment of the interaction between root endophytic microorganisms and their host plants. Two major directions merit further investigation: the regulatory factors governing endophytic microbial assembly and community establishment at the plant–microbe interface, as well as the assembly rules and symbiotic patterns formed by crop-associated microbial communities. High-throughput sequencing technology can be applied to identify the recruitment mechanisms of core endophytic microorganisms across diverse host species. Besides community-level investigations, further mechanistic studies are needed to decode the molecular crosstalk between endophytes and plant immune signaling. For instance, how endophytic strains fine-tune PTI and ETI responses via phytohormones, volatile organic compounds, and small RNAs remains largely unresolved for most crop-endophyte systems. Dissecting such multilayered signaling events will reveal how immunity serves as a host-selection filter and governs the functional output of endophytic communities. By evaluating plant–root endophytic interactions under various biotic and abiotic stresses, researchers can screen functional synthetic microbial consortia capable of activating plant systemic resistance and boosting crop productivity. Further large-scale microbiome surveys covering diverse native crop germplasm and locally adapted wild relatives are urgently required. Most current studies focus on a small set of model or widely cultivated crop accessions, whereas the endophytic microbiomes of wild plant relatives remain largely unexplored. Exploring these genetic resources may uncover valuable core endophytic taxa with superior stress-resistance and plant-beneficial traits. Meanwhile, advanced in-situ and live-cell imaging techniques should be further adopted to resolve the spatial colonization patterns of endophytes inside plant tissues. Such visual approaches help disentangle physical niche partitioning and microbe–microbe interactions within the host internal microenvironment, which cannot be fully captured by bulk sequencing alone. Moreover, future synthetic-consortia design should move beyond simple strain combination and prioritize the ecological compatibility among constituent members. Rational design needs to consider native soil background, host genotype, and fluctuating field environments to improve *in planta* persistence of inoculated microbes. Optimizing synthetic consortia for better ecological fitness will greatly narrow the gap between laboratory mechanistic findings and practical agricultural application of endophyte-based products. Similar to conventional crop breeding, plant microbiota serve as critical modulators of plant phenotypes. Targeted screening of favorable plant–microbe symbiosis facilitates the breeding of disease-resistant crop varieties. Therefore, harnessing microbiota to enhance crop disease resistance and yield is of great practical significance.

## Data Availability

No new data were created or analyzed in this study. Data sharing is not applicable to this article.
